# Supplementary material for: Impact of preoperative magnetic resonance imaging on surgery and eligibility for intraoperative radiotherapy in early breast cancer
Source: PLoS One. 2022 Oct 18;17(10):e0274385. doi: 10.1371/journal.pone.0274385 (PMC9578617; doi:10.1371/journal.pone.0274385)
Supplement: Attachment — Submitted filename: Response to reviewers 15.8.docx [file pone.0274385.s004.docx]

To

PLOS ONE Editorial Office

Dear Sir/Dr,

**Response to Reviewer’s Comments.**

Ms. No.: **PONE-D-21-32579**

Title: **IMPACT OF PREOPERATIVE MRI ON SURGERY AND ELIGIBILITY FOR INTRAOPERATIVE RADIOTHERAPY (IORT) IN EARLY BREAST CANCER**

Thank you very much for your comments to the above referenced manuscript and for consideration for publication in your respected journal. We highly appreciate the reviewers’ comments and have revised the manuscript according to the reviewer’s recommendation (see point-by-point reply enclosed herewith). We thank the reviewers for their valuable suggestions in improving the paper. The substantive changes have been addressed below with regards to each reviewers questions.

Below is the corrections/point-by-point reply to your comments.

Journal Requirements:

Additional Editor Comments:

Thank you for providing additional tumor/patient characteristics in Table 2. You were previously asked to address and emphasize the importance of these data as they may influence the results. We would like to invite you to add a paragraph in your results or discussion section to address this concern.

Author’s reply:

Thank you Editor for your constructive comments. We have now added several lines in the discussion to allude to the data in Table 2:

Line 394, 419 and 425.

I hope that these will be sufficient to address your concerns which we much appreciate.

We noticed that you provide the patient list with data as supplementary file. This should be mentioned in the material and methods section.

Author’s reply:

Thank you for your comment. We have now added a sentence within Materials and Methods: Data Collection and Analysis; Line 171.

Below is the response to the previous revision that we had done:

The reviewers raised important questions that I ask the authors to address and I emphasize the importance of describing the criteria for indicating MRI in this study in detail and the characteristics of patients in both study groups, with and without preoperative MR. Some pathological charateristics are described but there are some known criteria that favor the use of preoperative MRI due to the chance of impact on the conduct, I can mention Triple negative/HER-2/luminal B, High grade DCIS, Invasive lobular histology, dense breasts and young patients` tumors. Some of these characteristics of the population and subgroups were not provided and may influence the results.

Author’s reply:

Thank you for your constructive comment. In this study we did observe patients who were ER-/PR- as well as HER-2 negative and included them in the results particularly in Table 1. We have now added Table 2 which lists the Molecular Subtypes more clearly. We also noted that the majority of patients were in the category of BIRADS density B. We did not look at invasive lobular histology or DCIS in these patients as our focus as mentioned in methodology was on patients who had invasive ductal carcinoma. However, we did detect DCIS in some of the satellite lesions that were excised and this was discussed in the Discussion section. In addition, majority of our patients were in the 40-60 ages range, therefore to improve readability of this manuscript, we did not emphasise the younger age group. I hope that this addresses your comments and concerns.
Below is Table 2, which is located just after Table 1 in the manuscript.

**Table 2. Frequency of Molecular Subtypes in the study population**

| Subtype | N | % |
| --- | --- | --- |
| Luminal A | 46/54 | 85.1 |
| Luminal B | 5/54 | 9.2 |
| HER2+ | 2/54 | 3.9 |
| Triple Negative Breast Cancer | 1/54 | 1.8 |

*9 patients did not have complete hormonal information in order to fit the above subtypes
